# Natural-Compound Adjuvants Dismantle *Candida* Biofilms: Mechanisms, Design Rules, and Biofilm-Aware Pharmacology

**DOI:** 10.1007/s00284-025-04713-0

**Published:** 2026-01-12

**Authors:** Dang Anh Tuan, Jan Masak

**Affiliations:** Faculty of Food and Biochemical Technology, University of Chemistry and Technology, Prague, 166 28 Czechia

## Abstract

Device- and mucosa-associated candidiasis is difficult to cure because *Candida* biofilms shield cells from antifungals, leading to relapse and device failure. Standard treatment decisions are still largely guided by planktonic susceptibility tests, which poorly predict the drug exposure needed to clear mature biofilms. Here we synthesize evidence that natural-compound adjuvants can dismantle key biofilm defenses and outline design rules to rationalize biofilm-aware combination therapy. Across *Candida albicans*, non-*albicans* species and *Candida auris*, the most reproducible adjuvant effects fell into three themes: (1) reprogramming adhesion and morphogenesis, (2) disrupting membrane sterol homeostasis, and (3) weakening the extracellular matrix and efflux-mediated tolerance. When paired with standard antifungals, these actions frequently increase killing of established biofilms and reduce the exposures required for eradication. Local delivery approaches that concentrate actives at mucosal surfaces or device interfaces (nano- or surface-directed formulations) further improve intrabiofilm exposure while limiting systemic toxicity. We conclude that translation will require standardized biofilm assays, species-stratified testing and tighter links between biofilm pharmacology and clinically achievable exposure. The framework presented here is intended to help prioritize natural adjuvants and combinations most likely to benefit device-associated and mucosal candidiasis.

**Supplementary Information:**

The online version contains supplementary material available at 10.1007/s00284-025-04713-0.

## Introduction

*Candida* species are leading causes of mucosal disease and device-associated infections, and their clinical success hinges on the transition from planktonic cells to highly organized biofilms on biotic and abiotic surfaces—communities that are difficult to eradicate, resistant to conventional antifungals, and linked to excess morbidity and mortality [1]. Biofilm development proceeds from adhesion and initiation through maturation and dispersal, generating multilayered structures in which yeast, pseudohyphae, and true hyphae are embedded within an extracellular matrix enriched in β-1,3-glucans, mannans, proteins, lipids, and extracellular DNA [1, 2]. Within these mature communities, a β-1,3-glucan–rich matrix acts as a reactive diffusion barrier that sequesters antifungals, driving biofilm eradication thresholds (MBECs) far above planktonic MICs and explaining the frequent disconnect between MIC reports and real-world device-associated failures [3–6]. Beneath these phenotypes lies a deeply interconnected regulatory and signaling architecture: in *Candida albicans*, a small set of master transcription factors together with Ras1–cAMP–PKA and MAPK pathways integrates environmental cues to coordinate adhesin expression, morphogenesis, matrix biogenesis, and dispersal, while quorum-sensing molecules such as tyrosol and farnesol further tune biofilm development and virulence programs [7–11].

Biofilm-associated tolerance is multifactorial and extends beyond the matrix barrier. Shifts in membrane composition and ergosterol homeostasis, up-regulation of ATP-dependent efflux pumps—especially the ABC transporters Cdr1/Cdr2 and the MFS transporter Mdr1—and the presence of small persister subpopulations all contribute to survival at otherwise lethal drug concentrations [6, 12]. These layers help explain why standard monotherapy frequently fails on indwelling devices or heavily colonized mucosae and motivate a shift from simply “killing planktonic cells” toward multi-target strategies that dismantle biofilm-associated regulatory and tolerance networks [1]. Importantly, these networks are not uniform across species, including *C. albicans* and *C. tropicalis* display robust hypha formation, thick EPS-rich biofilms, and high biomass accrual, whereas *C. glabrata* remains yeast-locked with thinner, more sparsely structured biofilms and a more stress-tolerant, persistence-oriented lifestyle [2, 13]. The emerging multidrug-resistant pathogen *Candida auris* typically forms relatively thin but strongly adherent biofilms on skin, medical devices, and dry environmental surfaces, often in outbreak settings, with many isolates exhibiting high baseline MICs to azoles and, in some cases, reduced susceptibility to polyenes and echinocandins [14–16]. In *C. auris*, biofilm tolerance appears to rely predominantly on up-regulated efflux transporters, cell-wall and stress-response remodeling, and remarkable survival under disinfectant and desiccation pressure rather than on the classic hypha-centric virulence programs that dominate in *C. albicans* [14, 15].

Natural compounds—especially plant-derived phytochemicals—offer attractive multi-target leverage against these layered tolerance mechanisms. Molecules such as cinnamaldehyde, thymol/carvacrol, curcumin, and eugenol repeatedly attenuate adhesion and hyphal programs, destabilize membranes and ergosterol homeostasis, thin or chemically alter the extracellular matrix, and dampen efflux activity, with several combinations demonstrating synergy with licensed antifungals that compress the MIC–MBEC gap [17–19]. Advances in delivery science further enhance translational potential: nano- and liposomal formulations co-loading amphotericin B with curcumin or pairing micellar amphotericin with curcumin analogues improve potency while mitigating dose-limiting toxicity [20, 21]. Guided by these considerations, this narrative review pursues four primary objectives: (i) to identify compounds that modulate the expression or activity of key transcriptional regulators (e.g., UME6, EFG1, BCR1, TEC1) in *Candida* spp.; (ii) to summarize phenotypic disruption of biofilm formation, extracellular matrix production, and hyphal transitions across clinically relevant panels, including non-*albicans* species and *C. auris*; (iii) to examine impacts on virulence-associated traits such as adhesion and secreted hydrolases; and (iv) to assess synergistic combinations with approved antifungals that enhance biofilm-targeted efficacy and may help curtail resistance.

## Methodology

### Review Design and Scope

This article is a narrative review. Our aim is to integrate mechanistic and translational evidence on how regulatory targeting and natural-compound combinations narrow the MIC–MBEC gap in *Candida* biofilms. We prioritized studies with biofilm-aware endpoints (e.g., MBIC/MBEC or sessile time-kill), orthogonal readouts (microscopy or biophysical measurements alongside viability/biomass assays), and illustrative ex vivo or in vivo models. Rather than attempting exhaustive coverage or formal meta-analytic pooling, we sought to map mechanisms to practical antifungal endpoints and highlight exemplars where multiple lines of evidence converge.

### Information Sources, Eligibility, and Study Selection

We searched PubMed, Scopus, and Web of Science and complemented these with backward/forward citation chasing. Records were limited to English-language publications or English abstracts with sufficient methodological detail; preprints were consulted only when a peer-reviewed version was unavailable or materially identical. Reviews and guidelines were used selectively to orient the literature and terminology, whereas primary data drove all conclusions. The final comprehensive search across all sources was run on 23 August 2025. Eligible studies included in vitro (mono- or mixed-species) biofilms on clinically relevant materials, in vivo animal models, and clinical or observational work reporting biofilm-relevant outcomes. Exclusion criteria comprised non-*Candida* biofilms without a *Candida arm*, planktonic-only antifungal tests not linked to biofilm endpoints, opinion pieces lacking primary data, studies with insufficient methodological detail, and non-English records without adequate English summaries. Records retrieved from all sources were de-duplicated and screened in two passes (title/abstract, then full-text) against these criteria. Given the narrative design, screening and eligibility decisions were performed by the author with a confirmatory second pass; this review does not present a PRISMA flow diagram.

### Data Extraction, Quality Appraisal, and Evidence Synthesis

From each eligible study we extracted information on organisms and strains (clinical versus laboratory), biofilm models (substrate, media, shear, temperature, biofilm age), assay methods (biomass/viability assays, microscopy/biophysics, MBEC platforms), regulatory readouts (e.g., qPCR, RNA-seq, ChIP, quorum cues), virulence metrics (adhesion, secreted hydrolases), synergy metrics (FICI, Bliss, Loewe, HSA, ZIP), and key pharmacological parameters (MICs, MBECs, exposure regimens). Because formal risk-of-bias tools are not standardized for in vitro biofilm studies, we applied a domain-based checklist focusing on model validity, assay orthogonality, controls and comparators, replicate depth and statistics, mechanistic support, and reporting transparency; additional internal-validity considerations were applied to animal and clinical studies. Experimental heterogeneity in species/strains, biofilm age, surfaces, endpoints, synergy models, and reporting precluded meaningful meta-analysis. We therefore adopted a semi-quantitative narrative synthesis emphasizing the direction and consistency of effects across independent studies, typical magnitude ranges rather than single-point estimates, and triangulation across orthogonal readouts. A full description of database-specific search strings, detailed eligibility criteria, the list of extracted data items, the quality-appraisal checklist, and additional notes on how heterogeneity was handled is provided in Supplementary Methods S1.

## Biofilm Development and Architecture

### Stage Logic

**Fig. 1:**
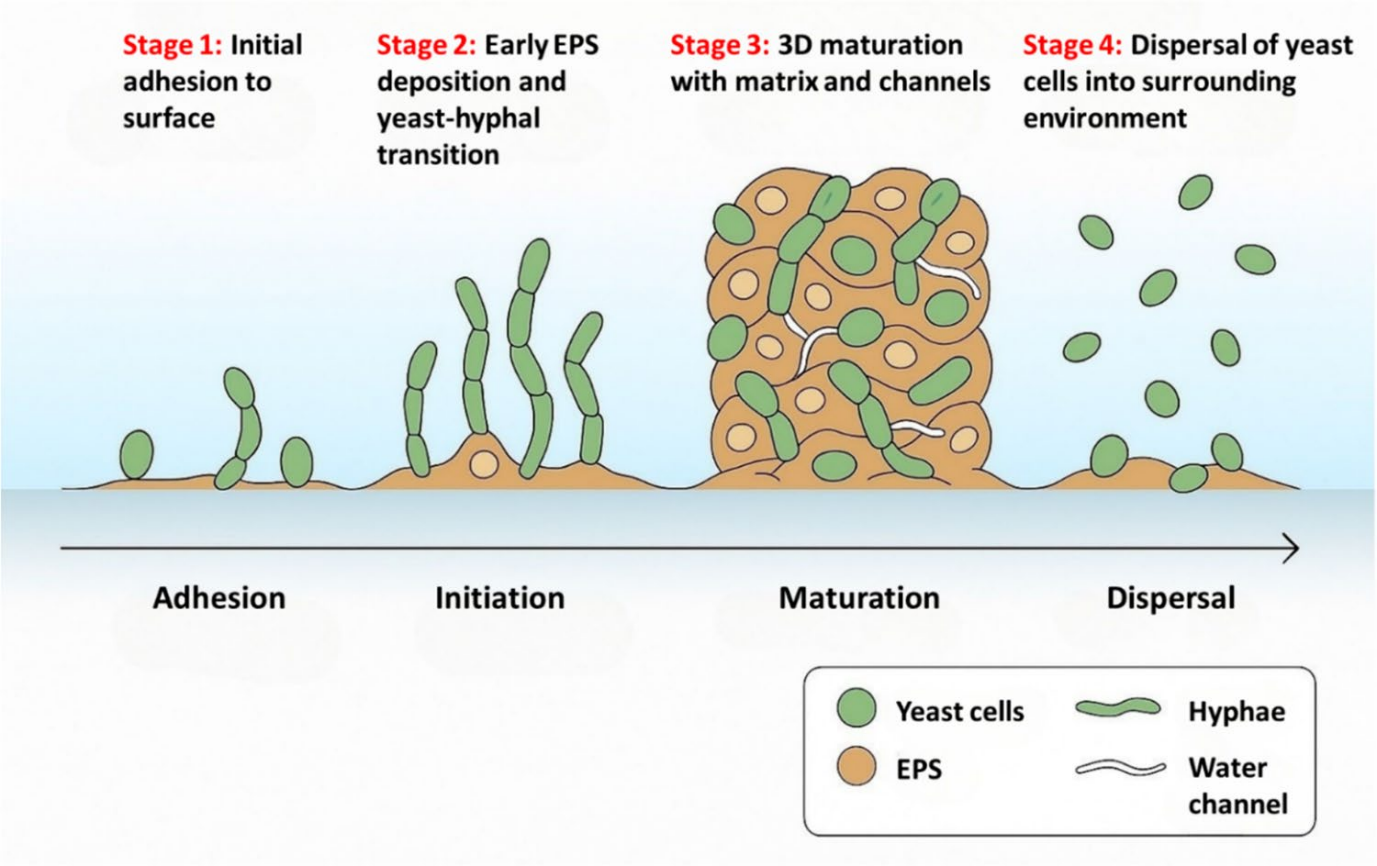
Sequential stages of *Candida albicans* biofilm development, illustrating the transitions from adhesion to dispersal

*Candida albicans* biofilm follows a staged program—adhesion → initiation (filament induction) → maturation (3D architecture + EPS) → dispersal—that is gated by environmental cues and quorum signaling. Early adhesion seeds a basal layer; initiation triggers yeast-to-hypha transition; maturation yields thick, channelled communities with peak tolerance; and dispersal releases blastospores that colonize new sites (Fig. 1). Authoritative stage frameworks from recent syntheses and mechanistic work support this four-step model [7].

### Adhesion and Initiation

Adhesion is mediated by Als-family adhesins (e.g., Als1/Als3) and Hwp1, which anchor cells to biotic/abiotic substrates and promote intercellular cohesion; genetic analyses and mutant phenotypes confirm their centrality in biofilm establishment [22–24]. Environmental and host cues—including CO₂, pH, nutrients—funnel into Ras1–cAMP–PKA and MAPK modules to commit cells to filamentation via regulators such as Efg1/Tec1, upregulating ALS/HWP adhesins and hyphal genes (ECE1, HYR1, HWP1, ALS3) [25, 26]. Quorum molecules fine-tune timing: tyrosol accelerates early filamentation, whereas farnesol antagonizes morphogenesis at higher density; host-metabolic signals (e.g., lactate) modulate morphogenesis/immune visibility and can reshape surface β-glucan exposure [8, 27].

### Maturation and Dispersal

Mature biofilms are multilayered, channelled structures that concentrate antifungal tolerance, immune evasion, and stress/efflux programs. Within these communities, gradients of nutrients/oxygen and localized physiology favor slow-growing or persister-like subpopulations and elevate ABC/MFS efflux activity, inflating eradication thresholds relative to planktonic MICs [28]. Dispersal releases yeast-form cells from biofilm surfaces; these dispersed cells frequently exhibit enhanced adhesion, invasiveness, and drug tolerance compared with planktonic counterparts, thereby bridging surface colonization to dissemination [29, 30].

### Architecture and Extracellular Matrix (EPS)

#### Thickness, Channels, Spatial Heterogeneity

Mature *Candida* biofilms are thick, vertically stratified communities with prominent water channels that generate steep intra-biofilm gradients (O₂, pH, nutrients) and spatially distinct physiological niches. Across models and substrates, *C. albicans* biofilms typically range from ~ 25 to > 450 µm in thickness; *C. glabrata* on catheter-like materials often forms thinner structures (~ 75–90 µm). These architectural features underpin drug tolerance by shaping diffusion and local pharmacodynamics [31–33].

#### Quantitative Composition and Variability

The matrix is a composite material averaging ~ 55% proteins, ~ 25% carbohydrates, ~ 15% lipids, and ~ 5% nucleic acids by dry mass in *C. albicans*, with species-, strain-, age- and surface-dependent variation. Polysaccharides organize predominantly as mannan–glucan complexes (MGCx); although β-1,3-glucan represents a minor fraction of total carbohydrate, it exerts outsized functional effects [6, 34–38]. Lipids encompass ergosterol-rich and sphingolipid fractions [38–41], and the nucleic-acid compartment is largely eDNA [32, 42].

#### Diffusion–Reaction Barrier and Drug Sequestration

Functionally, the EPS behaves as a reactive hydrogel: polymer networks slow diffusion and sequester antifungals, lowering the free drug fraction within the matrix. In *C. albicans*, matrix β-1,3-glucan is actively shuttled from wall synthesis into the EPS—so perturbing glucan processing or export diminishes tolerance. This glucan-centric sequestration contributes materially to the MIC–MBEC gap [6, 34–38].

#### Proteinaceous Scaffolding, Adhesins, and Hydrolases

Matrix proteins (~ 55%) provide cohesion and crosslinking and include adhesins (Als1/Als3, Hwp1), wall-remodeling enzymes, and secreted hydrolases (SAPs, phospholipases) that facilitate tissue invasion and immune modulation. Species-biased adhesin repertoires—e.g., Als/Hwp in *C. albicans* versus Epa families in *C. glabrata*—translate into measurable differences in mechanics and host-surface interactions [32, 38, 43, 44].

#### Lipids and Extracellular Vesicles (EVs)

The lipid (~ 15%) fraction—ergosterol, phospholipids, sphingolipids—organizes raft-like microdomains that influence antifungal partitioning. Biofilm-derived EVs traffic enzymes, virulence factors, and regulatory RNAs through the matrix and to host cells, shaping immune responses and stress adaptation. This helps explain why membrane-active adjuvants (e.g., thymol/carvacrol) often potentiate azoles: disrupting lipid domains/EV dynamics increases intramatrix drug bioavailability [38–41].

#### eDNA as an Ionic Cross-Linker

Although only ~ 1–5% by mass, eDNA strengthens the network via divalent-cation bridging (Ca^2^⁺/Mg^2^⁺), enhances early adhesion/cohesion, and can be targeted: DNase frequently reduces early biofilm biomass and, in some models, augments antifungal activity. Effects are stage- and strain-dependent and can be amplified in mixed-species communities [32, 42].

#### Assays and Readouts for Structure–Function Mapping

Robust inference requires multi-modal measurement: lectin-CLSM (ConA-mannans; WGA/CFW-chitin/β-glucan) and optical clearing for 3D architecture; HPAEC-PAD/GC–MS/NMR for carbohydrate chemistry; LC–MS lipidomics for lipid classes; DNase-sensitivity for eDNA contribution; and rheology/AFM for viscoelastic properties (see Table 1: “Common quantification/visualization assays”). Orthogonal quantification (e.g., CV and XTT ± CLSM z-stacks) and standardized parameters (biofilm age, inoculum, surface) are essential to compare across studies.

**Table 1:** Extracellular matrix (EPS) of *Candida* biofilms: composition, functions, assays, and species notes

EPS component	Typical proportion in *Candida* biofilms*	Representative constituents (examples)	Major functional roles in tolerance/virulence	Common quantification/visualization assays	Notes & species differences	Refs
Polysaccharides (β-glucans; mannans; minor chitin)	20–40% of matrix dry mass	β-1,3-glucan (± β-1,6 branches); α-mannans; mannan–glucan complex (MGCx); minor chitin	Physical scaffold; drug sequestration/partitioning; diffusion–reaction barrier; immune modulation (β-glucan masking/unmasking)	Lectin-CLSM (ConA-FITC for mannan; WGA/CFW for chitin/β-glucan); GC/NMR linkage; (HPAEC-PAD) monosaccharide profiling; FTIR	*C. albicans* matrix is ​​rich in MGCx; echinocandins act on the cell wall but matrix-related tolerance is maintained	[6, 34–38]
Proteins	40–60%	Adhesins: Als1/Als3, Hwp1; matrix enzymes: SAPs, phospholipases; wall-modifying enzymes; stress proteins; amyloid-like domains	Cohesion/adhesion; matrix cross-linking; tissue invasion (protease/PLB); immune evasion	SDS-PAGE/LC–MS proteomics; zymography; immunostaining; amyloid dyes (Congo red/ThT)	Adhesin repertoire differs by species*: C. albicans* (Als/Hwp1) vs *C. glabrata* (Epa)	[32, 38, 43, 44]
Lipids	10–20%	Ergosterol; phosphatidylinositol/serine; sphingolipids; lipid rafts; extracellular vesicle (EV) membranes	Hydrophobic barrier; carrier for hydrophobics; EV-mediated cargo delivery (enzymes, RNAs, toxins) within biofilms; affects ergosterol homeostasis and drug susceptibility	Folch/Bligh-Dyer extraction; LC–MS lipidomics; Nile Red/BODIPY; NTA/EM of EVs	EV cargo varies with stress and developmental phase; membrane phytochemicals (thymol/carvacrol) may increase azole sensitivity	[38–41]
Extracellular DNA (eDNA)	1–5%	Fungal/host-derived fragments; NET-associated DNA; cation-bridged lattices	Structural cross-linker (Ca^2^⁺/Mg^2^⁺); enhances adhesion/cohesion; contributes to tolerance; DNase can debulk early matrices	DNase sensitivity; PicoGreen/PI; qPCR; CLSM with DNA dyes	eDNA is most effective at early stages; mixed biofilm growth depends on eDNA	[32, 42]
Water & ions (microenvironment)	— (continuous phase)	H₂O; Ca^2^⁺, Mg^2^⁺, Na⁺, K⁺; small metabolites	Hydration & viscoelasticity; ion-bridging of polymers; shapes pH/O₂ gradients and drug diffusion	Microelectrodes (pH/O₂); FRAP of dextrans; rheology/AFM	Water channels & gradients are characteristic of mature biofilms (thickness hundreds of µm)	[33, 35, 45]
Physical microarchitecture (not a chemical component, but function-defining)	—	Water channels; vertical stratification; basal yeast layer + hyphal canopy	Diffusion pathways, convective exchange; niche partitioning (persister-like pockets); restrict immune access	CLSM z-stacks (live/dead, lectins); SEM/TEM; optical clearing + light-sheet	Typical thickness ~ 250–450 µm in *C. albicans* WT (depending on model); this architecture contributes to the MIC–MBEC gap	[31–33]

^*^ Proportions are approximate ranges aggregated from multiple models/conditions; the 55% protein, 25% carbohydrate, 15% lipid, 5% nucleic acid cutoffs are reported quantitatively for *C. albicans* and are often cited as reference standards (with variation by strain, surface, biofilm age) [38]

AFM, atomic force microscopy; CLSM, confocal laser scanning microscopy; ConA, concanavalin A; CFW, calcofluor white; EV, extracellular vesicle; FTIR, Fourier-transform infrared spectroscopy; GC, gas chromatography; HPAEC-PAD, high-performance anion-exchange chromatography with pulsed amperometric detection; MBEC, minimum biofilm eradication concentration; NTA, nanoparticle tracking analysis; ThT, thioflavin T; WGA, wheat-germ agglutinin

#### Translational Implications

The coupling of architecture (hundreds of µm; channel networks) and matrix chemistry (MGCx/β-glucan, protein crosslinks, lipids/EVs, eDNA) creates multicompartment PK/PD within the biofilm: peripheral, oxygenated zones may admit hydrophilic agents, whereas deeper lipid-rich or low-pH regions and efflux-enriched pockets diminish exposure and killing. Consequently, MBECs rise far above planktonic MICs. Therapeutic designs that remodel EPS (enzymatic or chemical), perturb membrane/lipid domains, and inhibit efflux, optionally coupled to targeted delivery (liposomes, nanoparticles), are most likely to compress the MIC–MBEC gap [46].

A consolidated summary of EPS composition, functional roles, assays, and species notes appears in Table 1.

## Regulatory and Signaling Networks

### Master Transcription Factors (TFs): An Integrated Biofilm Circuit

**Fig. 2:**
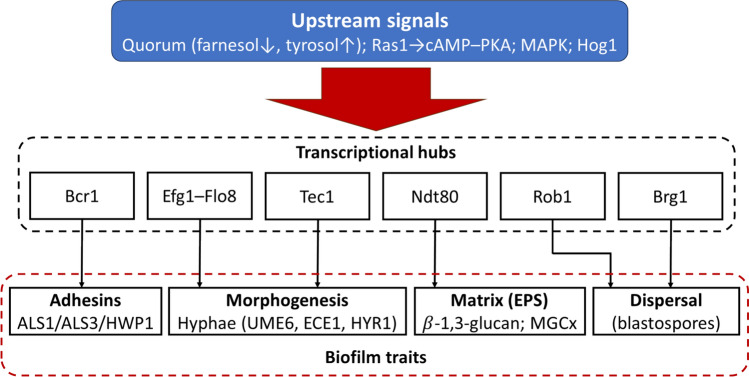
Simplified six-transcription-factor network linking upstream signals to core *Candida* biofilm traits. Host and environmental inputs—including quorum cues (farnesol, tyrosol), Ras1–cAMP–PKA, MAPK, and Hog1 pathways—converge on six transcriptional regulators (Bcr1, Efg1–Flo8, Tec1, Ndt80, Rob1, Brg1). Together, these hubs drive four major biofilm outputs: adhesin expression (ALS1, ALS3, HWP1), hyphal morphogenesis (UME6, ECE1, HYR1), extracellular matrix production (β-1,3-glucan; MGCx), and blastospore dispersal

A conserved circuit of master regulators—Bcr1, Efg1, Tec1, Ndt80, Rob1, Brg1—coordinates the major biofilm programs of *Candida albicans*: morphogenesis, adhesin expression, matrix biogenesis and dispersal (Fig. 2). Network-scale ChIP/perturbation work showed these TFs co-bind and cross-regulate each other and control > 1,000 target genes (≈1/6 of the genome), with a large fraction of targets occupied by two or more TFs, underscoring extensive combinatorial control. Mutations in individual nodes (e.g., *bcr1Δ, efg1Δ, ndt80Δ*) produce characteristic defects in adhesion, hypha formation, or mature biofilm architecture, and combined perturbations collapse biofilm development [10, 47, 48].

#### Therapeutic Implication

Because control is distributed across co-bound master TFs, single-pathway hits seldom abolish biofilms; dampening TF output together with downstream effectors (adhesins, matrix enzymes, efflux) is more likely to yield durable control.

### Quorum Cues: Farnesol Brakes, Tyrosol Accelerates

*Candida* uses small molecules to couple cell density to morphogenesis. Farnesol—a mevalonate-pathway isoprenoid—antagonizes filamentation and limits biofilm development; tyrosol—an aromatic alcohol—accelerates germ-tube formation at low density. In classic hypha-permissive conditions, 20 µM tyrosol increased germ-tube formation from ~ 5% to ~ 55% at 1 h and from ~ 15% to ~ 80% at 2 h, quantitatively demonstrating its pro-morphogenic role. Conversely, micromolar–submillimolar farnesol (≈3–300 µM) suppresses filamentation and thins early biofilms in vitro; mechanistically, farnesol inhibits Cyr1 (adenylate cyclase) to dampen cAMP–PKA signaling during hyphal induction. Recent reviews reaffirm farnesol/tyrosol as the best-validated quorum signals in *Candida*, with context-dependent effects during initiation versus maturation [49–51].

#### Therapeutic Implication

Exogenous or formulated quorum analogues can re-time morphogenesis/dispersal or serve as adjuvants (e.g., farnesol with azoles/echinocandins) to lower early-phase MBEC in models where timing dominates outcome.

### Signaling Pathways that Feed the TF Layer

#### Ras1–cAMP–PKA Axis

**Fig. 3:**
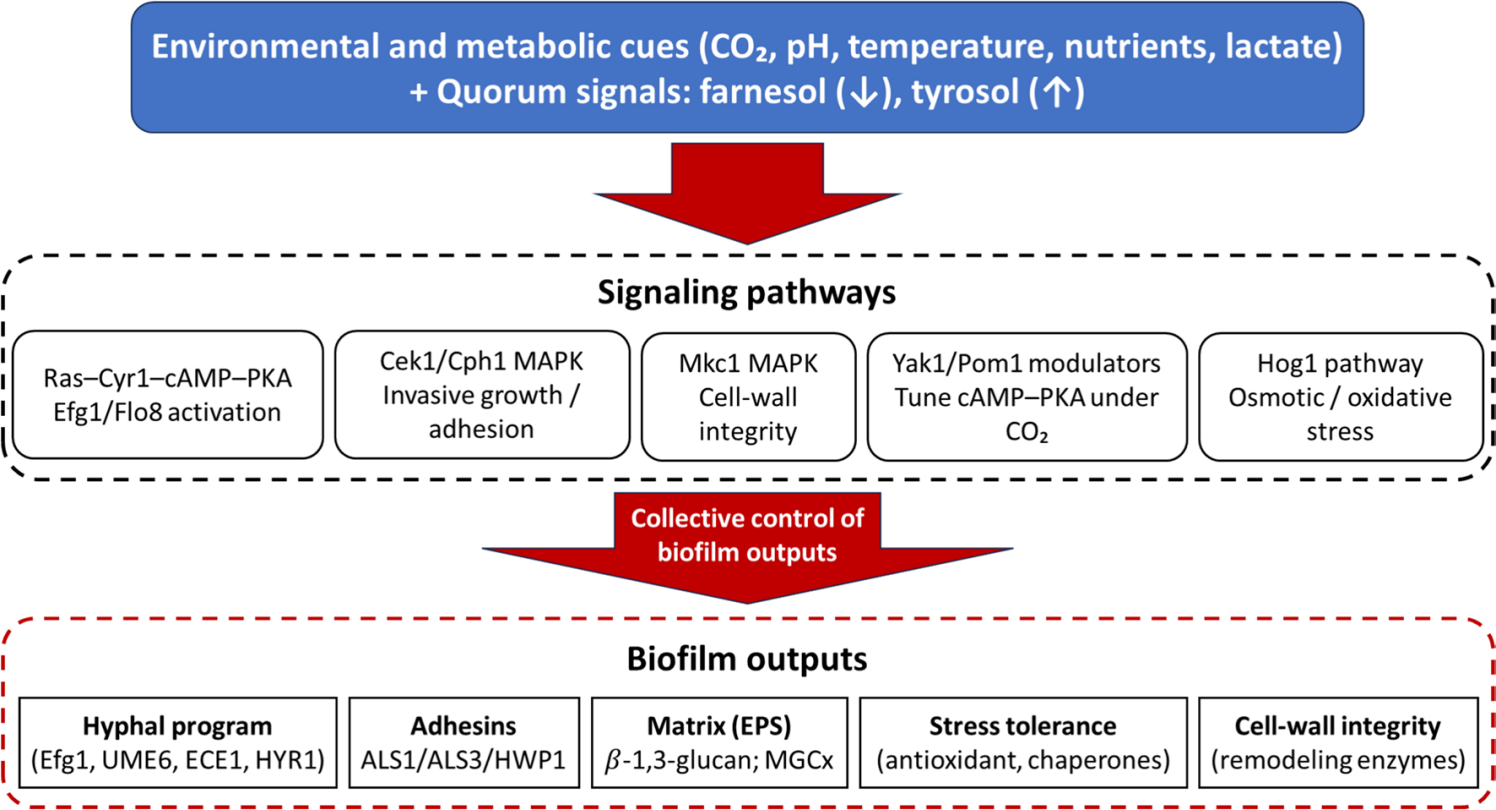
Environmental and stress signaling modules that impinge on *Candida* biofilm programs. Environmental and metabolic cues (CO₂, pH, temperature, nutrients, lactate) together with quorum signals (farnesol, tyrosol) are sensed through conserved pathways, including Ras–Cyr1–cAMP–PKA (activating Efg1/Flo8), Cek1/Cph1 and Mkc1 MAPK cascades, Yak1/Pom1 CO₂ modulators, and the Hog1 osmotic/oxidative-stress pathway. Collectively, these modules control key biofilm outputs such as hyphal program induction (Efg1, UME6, ECE1, HYR1), adhesin expression (ALS1, ALS3, HWP1), extracellular matrix production (β-1,3-glucan; MGCx), stress-tolerance programs (antioxidants, chaperones), and cell-wall integrity (remodeling enzymes)

Environmental cues (CO₂, pH, nutrients) converge on Ras1 → Cyr1 (cAMP) → PKA, which activates Efg1/Flo8 to drive hypha programs and adhesin expression (Fig. 3). Genetic and biochemical studies show Flo8 is indispensable for robust filamentation and virulence traits, while cAMP–PKA activity is both necessary and sufficient for hypha commitment across multiple media and temperature regimes [52, 53].

#### MAPK and Stress Modules

The Cek1/Cph1 MAPK pathway integrates cell-wall and mating cues; Mkc1 governs cell-wall integrity, and Hog1 mediates osmotic/oxidative stress adaptation. A quadruple MAPK-defective mutant (cek1 cek2 mkc1 hog1) remains viable but is hypersensitive to stress and antifungals, illustrating how these modules collectively secure robustness without being strictly essential for growth—yet they are pivotal for biofilm fitness under host-like stress [54, 55].

#### Emerging Regulators and Crosstalk

Upstream kinases such as Yak1 (DYRK family) and Pom1 modulate filamentation by tuning the cAMP–PKA pathway under host-relevant CO₂; dual deletion blocks filamentation across conditions, highlighting signal rerouting that preserves hyphal competence. Crosstalk nodes (e.g., Cpp1 phosphatase) interlink MAPK branches with morphogenic outputs, fine-tuning biofilm phenotypes [56, 57].

#### Therapeutic Implication

Combinations that attenuate Ras1–cAMP–PKA drive and erode cell-wall integrity buffering (Mkc1/Hog1) can collapse the hyphal scaffold and sensitize biofilms to licensed drugs, especially when paired with matrix-remodeling or efflux-damping strategies.

### Epigenetics and Non-coding RNA: Stabilizing (and Reprogramming) Biofilm States

Chromatin-level control (histone acetylation/deacetylation, Set3C and other remodelers) and chromatin accessibility dynamics modulate hypha- and biofilm-associated gene expression programs. Epigenetic tuning also intersects with antifungal resistance, influencing efflux and stress genes and shaping tolerance trajectories; histone deacetylase perturbation, for example, reprograms morphogenesis and drug responses. Non-coding RNAs (antisense/lncRNAs) are increasingly implicated in gating morphological switches and may buffer TF network noise during state transitions [58, 59].

#### Therapeutic Implication

Epigenetic adjuvants (e.g., selective KDAC inhibitors) could transiently re-sensitize biofilms by opening chromatin at repressed loci or dampening stress/efflux programs, but require careful targeting to avoid broad toxicity.

### Synthesis and Quantitative Design Rules

Taken together, these regulatory insights yield several quantitative design rules for antifungal adjuvant strategies. Control of *Candida* biofilms is distributed across six master transcription factors (and, context-dependently, a seventh with Flo8) that orchestrate more than ~ 1,000 co-bound targets, which helps explain why perturbing single nodes only partially reduces biofilms and favours multi-target approaches [48]. Timing of intervention is also critical: 20 µM tyrosol can increase early germ-tube frequency by roughly an order of magnitude, whereas 3–300 µM farnesol suppresses hypha formation, so exploiting this window improves early-phase intervention [49, 51]. Stress buffering is layered through MAPK, Hog1, and Mkc1 modules that maintain biofilm performance under host-like conditions; combinations that erode these buffers tend to synergize with agents that weaken the hyphal scaffold or extracellular matrix [55]. Finally, state memory exists at the epigenetic and non-coding RNA levels, such that brief modulation of chromatin or ncRNA circuits can reprogram morphology and drug susceptibility in otherwise tolerant biofilms [58].

## Virulence Traits and Antifungal Resistance

### Comparative Biology Across Major *Candida* Pathogens

#### Comparative Summary

Major *Candida* pathogens differ systematically in morphotype (hypha-competent vs yeast-locked), biofilm architecture (thick EPS-rich vs thin/sparse vs dense/invasive), and resistance style (matrix/efflux-dominant vs target-mutation–dominant vs mixed). These patterns explain why *C. tropicalis* is frequently severe and often azole-non-susceptible, whereas *C. albicans* and *C. glabrata* follow distinct architectural and drug-response logics. See Table 2 for head-to-head features; details and primary citations are summarized there [13, 60].

**Table 2:** Comparative features of major *Candida* species

Feature	*C. albicans*	*C. glabrata*	*C. tropicalis*	*C. auris*	Refs
Morphology	Yeast + hyphae + pseudohyphae	Yeast only	Yeast + hyphae	Yeast forms; no true hyphae, often aggregates or clumping cells	[15, 61–63]
Biofilm Type	Thick, structured, EPS-rich	Thin, sparse	Dense, invasive	Thin to moderately thick but strongly adherent biofilms on plastic/steel and skin; high tolerance and environmental persistence	[15, 61, 63–65]
Major Virulence Genes	*ALS, SAP, HWP, ECE*	*EPA, YAK1, SNQ2*	*SAP9/10, CDR1, ERG11*	Up-regulated ABC/MFS efflux pumps (CDR/MDR families), cell-wall remodeling and stress-response pathways rather than classic hyphal programs	[15, 62, 66, 67]
Antifungal Resistance	High via EPS + efflux pumps	High via target mutations	Moderate to high	Frequently multidrug-resistant with elevated MICs to azoles and occasional reduced susceptibility to polyenes and echinocandins; biofilms further inflate MBECs	[14, 15, 62, 67, 68]
Infection Models	Lethal in mouse/Galleria	Chronic persistence	Systemic invasion, necrosis	High-mortality bloodstream infections in outbreak settings; persistent colonization of skin and medical devices; resilient survival on abiotic surfaces	[14, 15, 63, 64, 69]
Host Interaction	Strong pro-inflammatory response	Immune evasion	High neutrophil recruitment	Efficient skin colonization and environmental survival in healthcare settings; resilience under disinfectant and desiccation stress; immune evasion and persistence at colonization sites	[14, 15, 63, 64, 68]

*Candida auris* warrants separate consideration because its biofilm and resistance profile is distinct from both *C. albicans* and *C. glabrata*. In vitro, *C. auris* typically forms relatively thin but strongly adherent biofilms on plastic and metal surfaces, as well as persistent communities on skin and dry environmental substrates in healthcare settings [61, 64, 65, 69]. These biofilms are highly tolerant to azoles, polyenes, and echinocandins, with sessile cells often requiring drug concentrations several orders of magnitude higher than planktonic MICs for comparable killing, and many clinical isolates already exhibit multidrug-resistant planktonic phenotypes before biofilm formation [15, 61, 62, 68]. Transcriptomic and functional studies further show that intermediate and mature *C. auris* biofilms up-regulate genes encoding ABC and MFS efflux pumps, cell-wall remodeling factors, and stress-response pathways, pointing toward an efflux- and stress-adapted mode of biofilm tolerance that only partially overlaps with the hypha-centric virulence programs typical of *C. albicans* [15, 62, 66]. These features suggest that membrane-active and efflux-modulating adjuvants—ideally delivered in surface-directed or device-focused formulations—may be particularly relevant for *C. auris* biofilms, but robust mechanistic and in vivo adjuvant data for this species remain sparse and represent a high-priority gap.

#### Clinical Note

Syntheses report ~ 55–60% all-cause mortality in invasive *C. tropicalis* infections, aligning with stress-tolerant biofilm phenotypes and frequent azole resistance [70, 71].

### Enzymology and host interaction

#### Secreted Hydrolases and Tissue Invasion

The Sap (secreted aspartyl proteinase) family (≈10 paralogs) and phospholipases constitute core invasive tools across *Candida* spp. Saps degrade host substrates, expose binding epitopes, and can act as adhesins via RGD/KGD motifs; phospholipases (notably PLB isoforms) disrupt host membranes and facilitate penetration. Contemporary reviews reinforce Sap/PLB activity as tightly coupled to biofilm maturation and mucosal damage [72, 73].

#### Hemolysins and Quantitative Signals

Phenotypic surveys report > 80% of clinical *C. albicans* isolates as strongly hemolysin-positive; controlled assays quantify RBC lysis ~ 29–44% depending on glucose availability, linking metabolic state to hemolytic output. Mechanistically, the hypha-associated toxin candidalysin (ECE1-derived) has been identified as a principal hemolytic factor, connecting morphogenesis to epithelial damage and immune activation [74–76].

#### Innate Immune Engagements Differ by Species

Mature *C. albicans* biofilms impair neutrophil function via matrix-induced signaling, reducing NET formation and killing; hyphae can physically escape macrophages, damaging phagosomal membranes and provoking inflammation. By contrast, *C. glabrata* often survives and replicates intracellularly with low macrophage damage and muted cytokine release—an evasion strategy consistent with its yeast-locked lifestyle and stress-tolerant physiology [77–80].

### Efflux, EPS Entrapment, and the MIC–MBEC Gap

#### Transporter Families and Exposure Control

Biofilm tolerance reflects reduced intracellular drug exposure via ATP-binding cassette (CDR1/CDR2) and major facilitator (MDR1) efflux systems, whose expression is frequently upregulated in azole-resistant isolates and in biofilm contexts. Network and expression studies (microarray/qPCR) consistently link pump overexpression to azole non-susceptibility across clinical cohorts [81, 82].

#### Matrix Sequestration of Azoles/Polyenes

Beyond pumps, β-1,3-glucan in the EPS binds and sequesters antifungals; disrupting glucan delivery to the matrix (e.g., Bgl2, Phr1, Xog1 pathway) restores susceptibility—pinpointing a extracellular, physical–chemical barrier that operates independently of cell-intrinsic resistance. This explains why strategies that degrade or remodel matrix glucans can potentiate conventional drugs [6, 83].

#### Quantifying the Gap

**Fig. 4:**
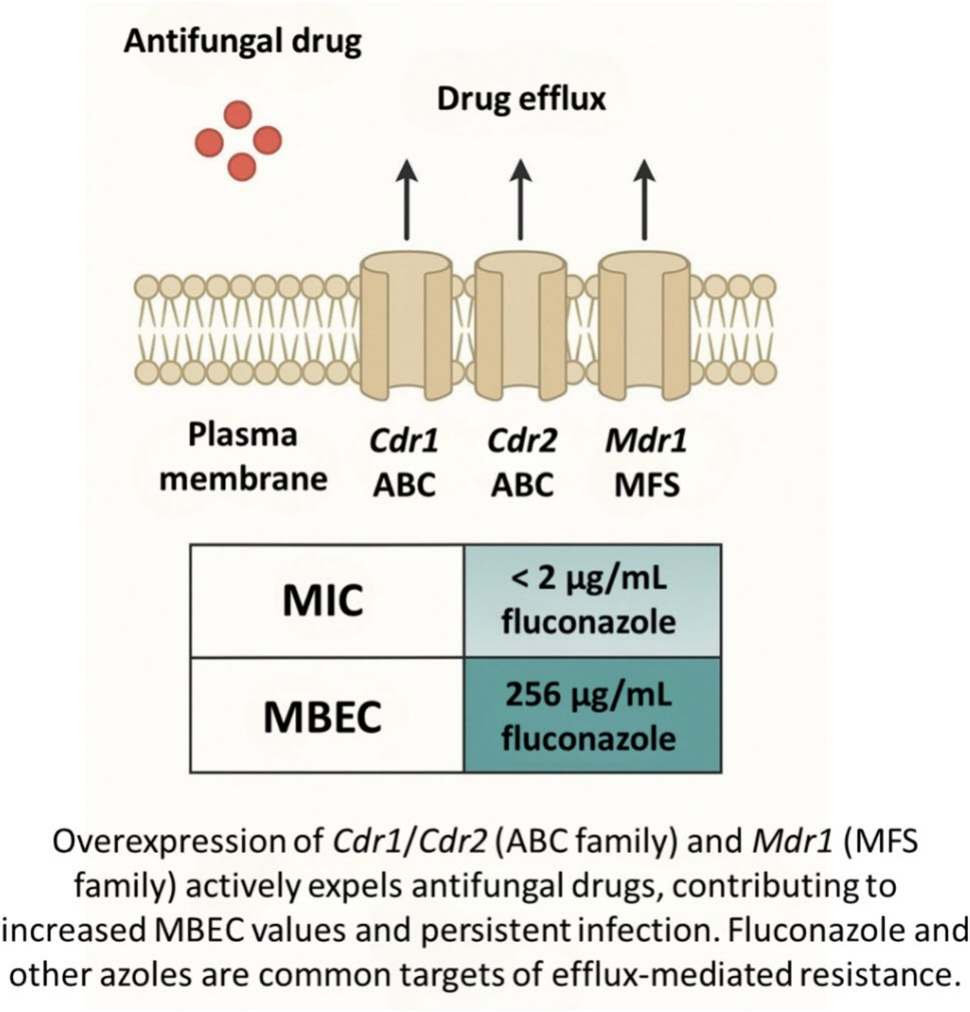
Antifungal resistance mechanisms in *C. albicans* biofilms mediated by membrane-bound efflux transporters (*CDR1*, *CDR2*, *MDR1*). Created by author, based on data from [86, 87]

Across strong biofilm-forming *C. albicans* isolates, planktonic fluconazole MICs typically fall within ≤ 0.125–8 µg/mL, yet MBECs against mature biofilms frequently jump to ≥ 64 µg/mL and can reach ≥ 256 µg/mL, i.e., 64–1,024-fold higher than planktonic benchmarks (representative ranges across multiple cohorts and models). Foundational phase-resolved work similarly reported biofilm MIC > 256 µg/mL for fluconazole in mature communities, despite comparable biomass among pump mutants and parent strains—highlighting multi-layered tolerance (matrix + physiology + pumps) [84, 85]. This exposure gap is illustrated in Fig. 4.

#### Implications for Therapy

Because tolerance is multifactorial, successful regimens combine pump attenuation (direct inhibitors or transcriptional down-tuning), matrix remodeling (enzymatic or chemical glucan/eDNA targeting), and enhanced delivery (e.g., liposomal or nanoparticle carriers), often yielding order-of-magnitude reductions in MBEC when paired with azoles or echinocandins [83].

## Natural Compounds and Mechanistic Disruption

### Mechanism Spectrum

Rather than considering each molecule in isolation, the best-characterised plant-derived agents can be organized into three mechanistic clusters that converge on complementary control points along the *Candida* biofilm lifecycle.

The first cluster comprises **regulatory-biased phenylpropanoids** such as cinnamaldehyde (CA) and eugenol (EUG). At sub-inhibitory concentrations, these agents attenuate early adhesion and hyphal initiation, with RT-qPCR data showing consistent down-regulation of adhesin and hypha programmes (ALS1/ALS3/HWP1, CPH1, SAPs, PLB1) together with curtailed filamentation and delayed biofilm maturation. CA and optimized CA analogues remodel three-dimensional biofilm architecture and can prevent > 90% biofilm formation at 50 μg/mL for α-methyl and 4-methyl CA, accompanied by gene-level decreases in UME6/ECE1/RBT5/UCF1 and reciprocal YWP1 up-shifts [88].

A second cluster is formed by **membrane-active phenolic monoterpenes**, typified by thymol and carvacrol (THY/CAR). These compounds disrupt fungal membranes and ergosterol homeostasis, elevate intracellular ROS and display broad fungicidal activity across both azole-susceptible and azole-resistant isolates. When combined with azoles, THY/CAR frequently deliver pharmacodynamic synergy, with most strains exhibiting FICI values in the approximate 0.25–0.75 range under biofilm-aware conditions [89, 90].

The third cluster encompasses **polyphenolic adjuvants** such as curcumin (CUR). Curcumin suppresses adhesion and secreted virulence-enzyme activities and acts as a potentiator of amphotericin B (e.g. FICI = 0.5 with a four-fold reduction in AMB MIC in clinical isolates), while nano-formulations further reduce biofilm thickness and adhesion in CLSM/SEM analyses [91, 92].

**Fig. 5:**
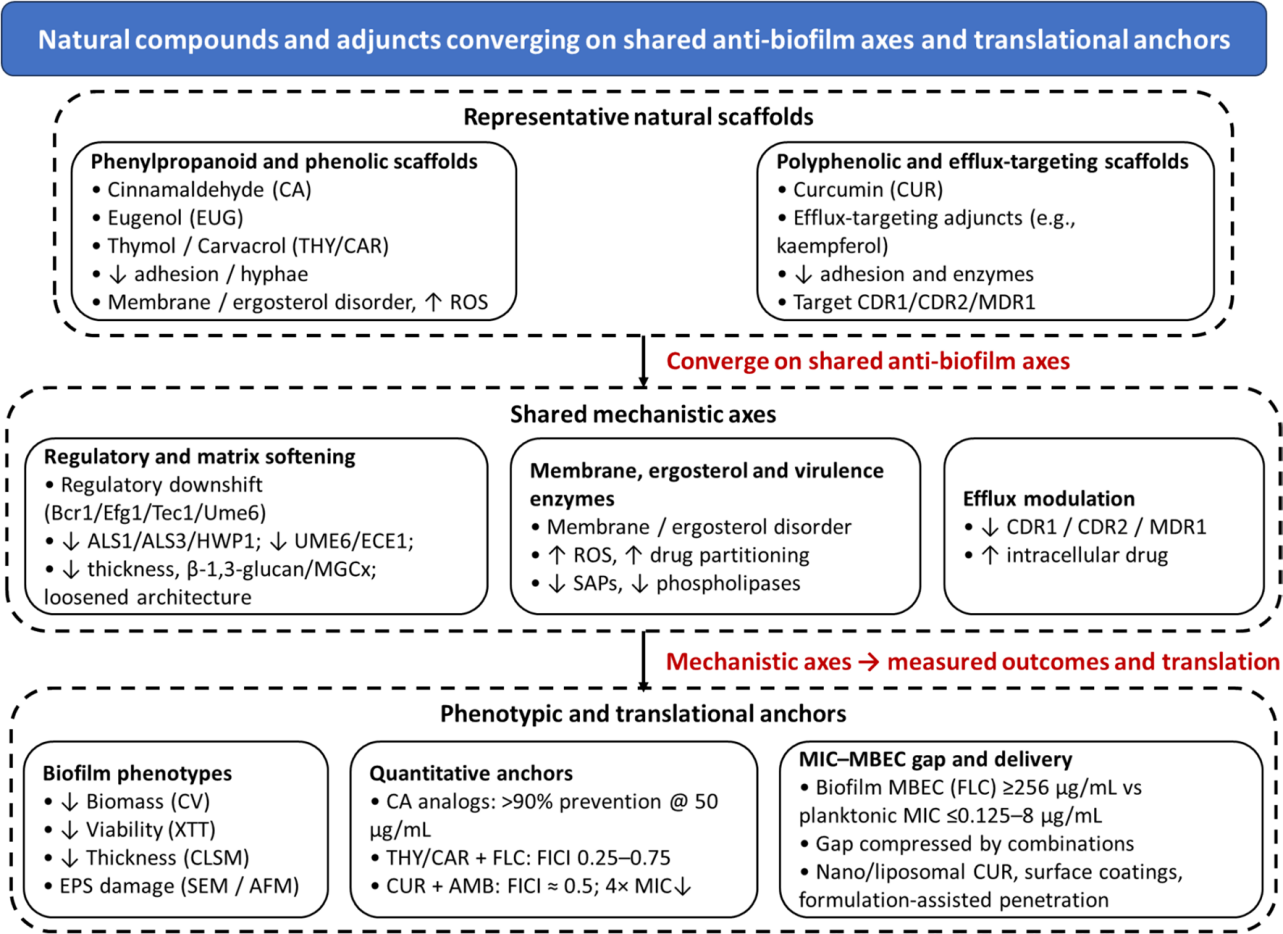
Natural-compound scaffolds converging on shared anti-biofilm axes and translational anchors. Phenylpropanoid and phenolic scaffolds (cinnamaldehyde [CA], eugenol [EUG], thymol/carvacrol [THY/CAR]) primarily downshift adhesins and hyphal programs, soften the extracellular matrix and perturb membranes/ergosterol homeostasis, often increasing reactive oxygen species. Polyphenolic adjuvants (curcumin [CUR] and efflux-targeting flavonoids such as kaempferol) suppress adhesion and virulence-enzyme output and dampen efflux/stress responses, while being amenable to delivery-enhancing formulations. These mechanistic axes converge on shared biofilm phenotypes (↓biomass/viability, ↓thickness, EPS damage) and yield quantitative gains in combination with standard drugs, such as CA analogues preventing > 90% biofilm at 50 μg/mL, THY/CAR plus fluconazole frequently showing synergy (FICI ≈ 0.25–0.75), and CUR plus amphotericin B achieving FICI ≈ 0.5 with a four-fold MIC reduction. The schematic emphasizes grouped mechanisms and shared control points rather than a one-to-one mapping between every compound and every readout

Taken together, these three clusters—regulatory-biased phenylpropanoids, membrane-active monoterpenes and polyphenolic adjuvants—illustrate how natural compounds can multi-hit regulatory circuits, membranes and virulence outputs to weaken *Candida* biofilms. A visual mechanism montage is provided in Fig. 5, and a structured evidence summary, including MIC values, biofilm inhibition and mechanistic highlights for representative phytochemicals, appears in Table 3.

**Table 3:** Representative phytochemicals active against *C. albicans* biofilms, organized by mechanistic cluster

Compound	Plant Source	MIC (µg/mL)	Biofilm Inhibition	Mechanism Highlights	Refs
Cinnamaldehyde	*C. verum* (Cinnamon)	~ 64	Early biofilm and EPS	Cluster 1 (regulatory-biased phenylpropanoid); downregulates ALS3/HWP1, softens matrix	[93]
Eugenol	*S. aromaticum* (Clove)	50–100	Matrix disruption	Cluster 1; decreases ALS1/ALS3/HWP1, SAPs, PLB1; impairs adhesion and biofilm maturation	[94]
Thymol	*T. vulgaris* (Thyme)	~ 100	Synergistic (with azoles)	Cluster 2 (membrane-active phenolic monoterpene); ROS↑, membrane/ergosterol disruption	[95]
Curcumin	*C. longa* (Turmeric)	32–128	50–70% biomass ↓	Cluster 3 (polyphenolic adjuvant); ROS-mediated effects, adhesion/virulence-enzyme suppression, synergy with amphotericin B	[95]

### Regulatory Targeting

Across the three mechanistic clusters, natural compounds consistently modulate the transcription factor–pathway layer that gates biofilm development, rather than abolishing it outright. Within the regulatory-biased phenylpropanoid cluster (cluster 1), eugenol congeners drive coherent down-regulation across ALS1/2/3/9, HWP1 and CPH1 (MAPK-linked), together with SAPs and PLB1, in line with their anti-adhesion and anti-hypha phenotypes. Cinnamaldehyde analogues similarly reduce UME6 and hypha-associated transcripts such as ECE1 and UCF1, while shifting expression toward YWP1 (yeast-associated programme), a transcriptional signature compatible with dampening of the Efg1/Tec1 arm [88].

For the membrane-active phenolic monoterpenes (cluster 2; thymol/carvacrol), direct TF-level datasets are fewer, but their ability to perturb ergosterol synthesis and membrane order is expected to indirectly attenuate cAMP–PKA/MAPK outputs that normally sustain Efg1/Tec1 activity. In azole-synergy exemplars catalogued in the essential-oil literature, membrane targeting by THY/CAR frequently coincides with lower expression of key TF-controlled modules and improved azole performance [89, 96].

Within the polyphenolic adjuvant cluster (cluster 3), curcumin often reprograms virulence-enzyme activity and stress responses without requiring consistent gene-level knockdown in all contexts, suggesting post-transcriptional or membrane-proximal control that nonetheless converges on reduced biofilm fitness [97].

Taken together, these observations indicate a shared regulatory phenotype across clusters: partial suppression of EFG1/TEC1/BCR1/UME6 modules—proxied by decreases in ALS/HWP/ECE programmes and yeast-leaning markers such as YWP1—rather than complete transcriptional silencing [98].

### Phenotypic Disruption of Biofilm Structure and Function

At the biofilm-phenotype level, cluster-level patterns are more informative than compound-by-compound descriptions. Phenylpropanoids in the regulatory-biased cluster (cluster 1) preferentially interfere with early-phase biofilm establishment: cinnamaldehyde analogues achieve > 90% prevention of biofilm formation at 50 μg/mL while planktonic MICs remain ≥ 200 μg/mL, underscoring a bias toward biofilm-selective interference; at sub-MICs, eugenol inhibits both single- and mixed-species *Candida* biofilms in vitro [99, 100]. These effects translate into lower biomass and viability in standard crystal-violet and metabolic assays, consistent with the transcriptional dampening of adhesin and hypha programmes described in Sect. "Regulatory Targeting".

Membrane-active monoterpenes and polyphenolic adjuvants in clusters 2 and 3 exert more pronounced effects on mature biofilm architecture. Confocal and scanning electron microscopy consistently show significant thinning and architectural loosening after phenolic treatment, with natural-compound exposure yielding shallower biofilms and disrupted three-dimensional organisation. Curcumin-based nano-systems, in particular, reduce biofilm thickness on silicone and other device-like surfaces in both short- and longer-term assays (p < 0.02) and disrupt continuity of the extracellular polymeric substance (EPS) layer [91, 101].

Across clusters, multiple studies document EPS ultrastructure damage and altered live/dead distributions. CA/EUG/CUR formulations often increase the proportion of red-staining (compromised) cells and fragment the matrix on SEM/CLSM, indicating matrix softening that complements the upstream regulatory effects [102]. In aggregate, these findings support a convergent phenotype in which natural-compound clusters lower biomass and viability, decrease biofilm thickness and compromise EPS integrity, thereby weakening the physical and functional barrier that underpins biofilm tolerance.

### Virulence and Host–Interaction Impact

At the virulence level, compounds in the regulatory-biased phenylpropanoid cluster (cluster 1; eugenol congeners and cinnamaldehyde analogues) and the polyphenolic adjuvant cluster (cluster 3; curcumin) converge on adhesion, invasion and tissue damage. Across model systems, CA/EUG exposure down-shifts canonical adhesin and hyphal programmes (ALS1/ALS3/HWP1 and UME6/ECE1), and these transcriptional changes track with measurable losses in surface adhesion, shallower tissue invasion and reduced hypha-driven penetration [88]. Curcumin further decreases secreted aspartyl proteinase and phospholipase activities, mitigating enzymatic tissue-damage signatures and weakening the overall virulence profile.

By thinning biofilms, softening the matrix and disordering membranes, these clusters are also poised to modulate host responses: reductions in hyphal burden and candidalysin-linked injury are expected to lessen inflammatory triggers while simultaneously improving immune access to embedded cells. Experimental work in related settings suggests that matrix loosening and lower hyphal density alleviate matrix-mediated impairment of neutrophil functions, including extracellular trap formation and effective phagocytic engagement [77]. Taken together, virulence- and host-interaction effects complement the regulatory and structural disruptions outlined in Sects. "Mechanism Spectrum"–"Phenotypic Disruption of Biofilm Structure and Function", supporting the view that natural-compound clusters weaken *Candida* biofilms by multi-level interference rather than a single-point hit.

### Synergy with Standard Antifungals

Mechanistically, all three clusters lend themselves to combination use with standard antifungals, but the strongest quantitative evidence currently comes from the membrane-active monoterpenes (cluster 2) and the polyphenolic/alkaloid adjuvants (cluster 3). Thymol and carvacrol, as prototypical cluster-2 agents, frequently yield synergy with fluconazole, with FICI values in the range of 0.25–1.0 in susceptible backgrounds and 0.29–0.75 in azole-resistant isolates [90]. These interactions are consistent with membrane fluidisation and ergosterol-pathway perturbations increasing intra-biofilm drug availability and potentiating fluconazole’s intrinsic activity.

Within cluster 3, curcumin exemplifies polyen potentiation: CUR plus amphotericin B achieves FICI = 0.5 with a four-fold reduction in AMB MIC, and co-loaded CUR/AMB microparticles further suppress adhesion and biofilm readouts, illustrating how delivery-enabled co-localisation can amplify synergy [92]. Efflux-targeting phytochemicals extend this logic; kaempferol combined with fluconazole down-regulates CDR1/CDR2/MDR1 (qPCR) with demonstrable synergy in azole-resistant isolates, while broader surveys highlight alkaloids such as tetrandrine as pump modulators that lower azole MICs and display antibiofilm effects. Marine-natural-product reviews likewise identify indole and isoquinoline scaffolds with anti-*Candida* potential—often efflux- or membrane-directed—although translational datasets remain at an early stage [103, 104].

At the biofilm state, baseline MBECs for mature *Candida* biofilms can exceed 256 µg/mL for fluconazole (versus planktonic MICs ≤ 0.125–8 µg/mL), i.e. two to three orders of magnitude higher. Phenolic and alkaloid adjuvants from clusters 1–3 collectively compress this gap by softening the EPS, perturbing membranes and reducing efflux, thereby improving drug penetration, retention and local effective concentration within the biofilm [105].

### Integration and Design Principles

Viewed together, the three mechanistic clusters suggest that effective regimens should act on two complementary fronts: they dampen the regulatory programmes that drive adhesins and hyphae (cluster-1 phenylpropanoids and selected cluster-3 agents) while weakening the material defences of the biofilm—its EPS and membranes (clusters 2 and 3). Biofilm selectivity can be exploited: cinnamaldehyde, eugenol, thymol and curcumin often impair biofilms more than planktonic cells at equivalent exposures, so early-phase dosing offers an opportunity to pre-empt maturation, when tolerance peaks and MBEC–MIC gaps are maximal [99, 100].

Delivery acts as a force multiplier across clusters. Nano- and micro-formulations that co-localise actives within matrix channels and at the biofilm–device interface amplify CLSM/SEM readouts (thinner films, lower viability, fragmented EPS) and are particularly suited to device-surface applications, where sustained local concentrations can be maintained without systemic toxicity [91, 102].

Finally, synergy should be planned and verified rather than assumed. A practical design rule is to pair agents that (i) increase free drug in the matrix (membrane fluidisers and ergosterol modulators), (ii) knock down efflux capacity, or (iii) disrupt EPS architecture, and then systematically evaluate the interaction with FICI (checkerboard, ideally including sessile conditions) and, where possible, MBEC under biofilm-aware protocols [90, 104]. Such mechanism-informed combination testing can help prioritise natural-compound adjuvants that deliver genuine biofilm-level gains beyond standard monotherapy.

## Synergistic Strategies to Close the MIC–MBEC Gap

### Mechanistic Complementarity: Pair Hits to Increase Free Drug and Collapse Tolerance

**Fig. 6:**
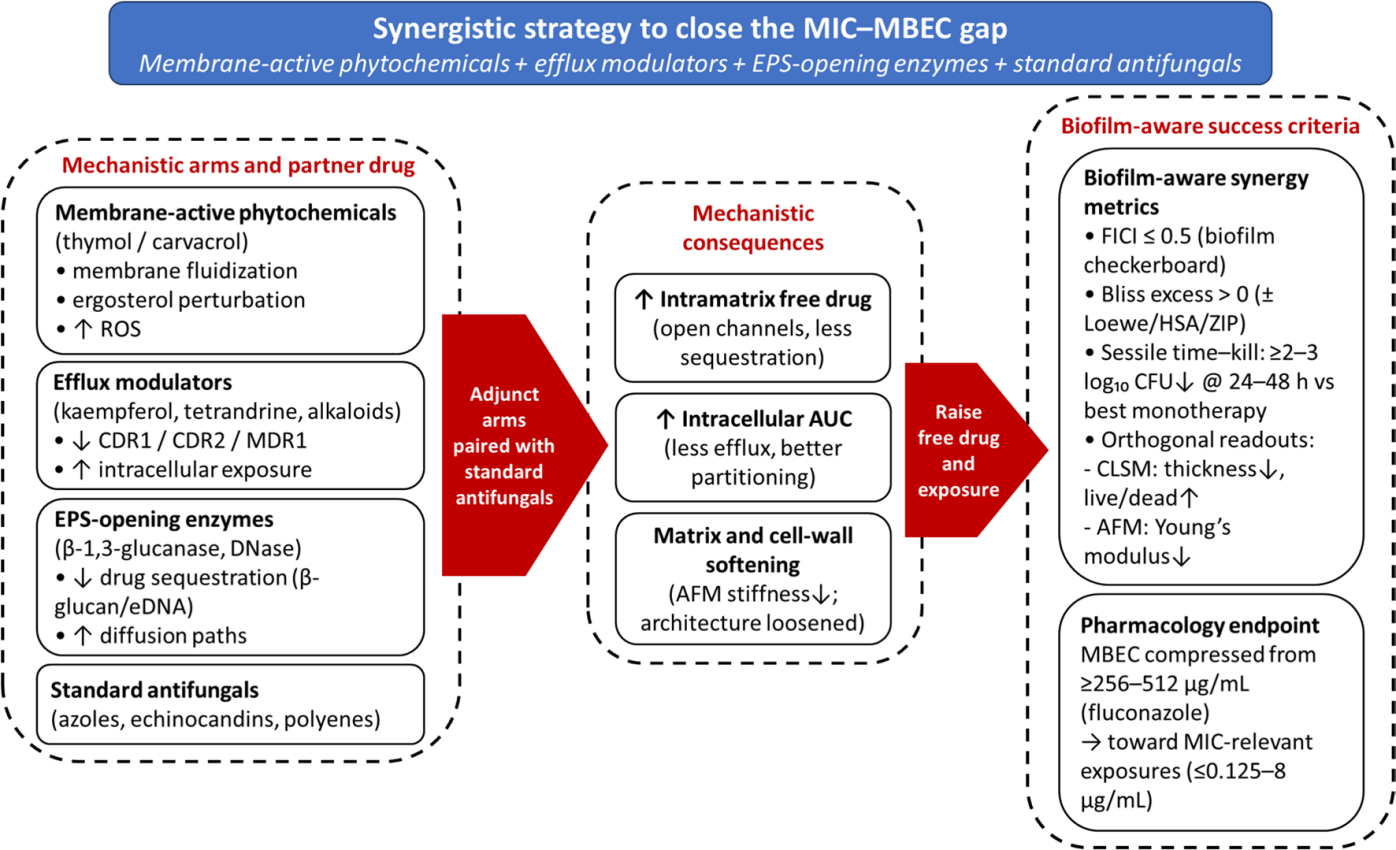
Synergistic strategies to close the MIC–MBEC gap. Three complementary adjunct arms—membrane-active phytochemicals (e.g. thymol/carvacrol), efflux modulators (e.g. kaempferol, tetrandrine), and EPS-opening enzymes (β-1,3-glucanase, DNase)—are paired with standard antifungals (azoles, echinocandins, polyenes) to raise intramatrix free drug, increase intracellular exposure (AUC), and soften matrix/cell-wall mechanics in mature *Candida* biofilms. Biofilm-aware success criteria include robust synergy on checkerboards (FICI ≤ 0.5 with positive Bliss excess or equivalent model-based evidence), ≥ 2–3 log₁₀ improvements in sessile time–kill versus the best monotherapy, concordant structural/biophysical disruption on CLSM and AFM, and pharmacologic compression of MBECs from typical high bands (≥ 256–512 µg/mL for fluconazole) toward planktonic MIC-relevant exposures. The schematic emphasizes mechanistic complementarity and integrated endpoints rather than detailed assay protocols

A practical way to compress the planktonic-to-biofilm exposure gap is to stack complementary mechanisms: (i) Membrane-active phytochemicals (thymol/carvacrol) fluidize membranes and perturb sterol homeostasis, which improves azole partitioning into cells and matrix channels; (ii) efflux modulators increase intracellular residence time of azoles; and (iii) EPS-degrading enzymes (β-1,3-glucanase, DNase) open diffusion paths and reduce drug sequestration. Canonical checkerboard studies show carvacrol + fluconazole synergy with FICI ≈ 0.31 in *Candida*, while broader screens report eugenol and cinnamaldehyde synergizing with fluconazole against *C. albicans* biofilms; thymol’s interaction varies by strain and assay (synergy to “no interaction”), arguing for matrix-aware testing designs. β-1,3-glucanase reduces established *C. albicans* biofilm biomass by ~ 56% and sensitizes to both fluconazole and amphotericin B; DNase likewise potentiates antifungals in sessile assays. Together, these moves raise free drug (matrix) and intracellular exposure (cell), collapsing the biofilm tolerance ensemble that drives MBEC ≫ MIC (often ≥ 256–512 µg/mL for fluconazole vs ≤ 0.125–8 µg/mL MIC) [106–109] (Fig. 6).

### Quantitative Endpoints and Success Criteria Under Biofilm-Aware Conditions

To ensure that “true synergy” is not confounded by growth phase, inoculum effects, or matrix-related artefacts, biofilm-focused combination studies should go beyond classical planktonic checkerboards and report multiple, orthogonal metrics under bona fide biofilm conditions. In practical terms, this means combining synergy metrics, sessile killing kinetics, structural/biophysical readouts, and pharmacological benchmarks that together define what “success” looks like for *Candida* biofilm combinations.

#### Synergy Metrics

Checkerboards should be performed under biofilm-aware conditions (e.g., mature biofilms on relevant surfaces) with conventional FICI thresholds (synergy ≤ 0.5, indifference 0.5–4, antagonism > 4) and explicit reporting of whether inputs are planktonic MICs or sessile MIC/SMIC values. When possible, these matrix-based checkerboards should be complemented with model-based analyses such as Bliss independence (for independent-action drugs) or Loewe additivity (for same-target drugs); model choice matters and can bias synergy scores when dose–response slopes are not fitted appropriately [110, 111]. As a pragmatic success criterion, we suggest that candidate combinations should achieve FICI ≤ 0.5 on checkerboard or Calgary-type biofilm platforms, together with positive Bliss excess (or equivalent model-based evidence of synergy) across at least three concentration pairs, and should avoid single-point claims.

#### Sessile Killing Kinetics

Static eradication endpoints alone can be misleading. We therefore recommend adding sessile time–kill experiments (0–48 h) that track CFU or metabolic readouts (XTT/MTT) for combinations versus monotherapies under mature-biofilm conditions. Several anti-*Candida* pairs that appear merely additive on checkerboards reveal ≥ 2–3 log₁₀ CFU deficits relative to the best single agent in sessile time–kill assays [112, 113]. As a working benchmark, combinations that achieve a ≥ 2–3 log₁₀ CFU reduction versus the most active monotherapy at 24–48 h, with area under the killing curve (AUC) reported to capture overall exposure–response, can be considered promising.

#### Phenotype and Mechanics

Structural and biomechanical changes provide an additional, orthogonal lens on biofilm disruption. We recommend pairing CLSM live/dead imaging and thickness measurements with AFM-derived Young’s modulus and adhesion forces to quantify matrix softening and cell-wall weakening during combination therapy; AFM measurements at 37 °C are particularly informative for capturing hyphal mechanics and drug-induced softening in *Candida* biofilms. For susceptibility benchmarks, Calgary biofilm devices or equivalent SMIC₈₀-based platforms help standardize biofilm age and readouts [84, 114]. As a practical target, mature biofilms exposed to successful combinations should show on the order of ≥ 30–50% decreases in thickness (CLSM) and Young’s modulus (AFM), consistent with genuine matrix softening rather than superficial biomass effects.

#### Pharmacology and MIC–MBEC Compression

Ultimately, quantitative endpoints should speak to the MIC–MBEC gap and to clinical feasibility. For azoles and polyenes, a useful pharmacological benchmark is compression of biofilm eradication requirements from high-hundreds (≥ 256–512 µg/mL or higher) toward clinically achievable exposures, ideally within the same order of magnitude as planktonic MICs [115]. Combinations that substantially reduce MBECs while maintaining or improving safety profiles (e.g., via dose-sparing effects) are more likely to translate to device- or mucosa-associated infections.

#### Brief Methods Note for Reproducibility

To facilitate comparison across laboratories, we recommend Calgary biofilm devices or equivalent 96-well sessile platforms for checkerboards (SMIC₈₀ readouts), time–kill studies on 24–48 h “mature” biofilms, and CLSM plus AFM as orthogonal structural/biophysical endpoints. Synergy should be judged using both FICI and at least one explicit model-based metric (such as Bliss or Loewe) to mitigate bias from arbitrary cut-offs and non-linear dose–response relationships [84, 116]. Together, these quantitative guardrails provide an operational definition of what “success” looks like in the Results sections of biofilm-targeted combination studies.

### Formulation Leverage: Put More Drug where it Matters

Formulation strategies can be used to place natural-compound adjuvants and partner antifungals precisely in the compartments where *Candida* biofilms are most vulnerable. Nanocarriers such as liposomes and PLGA/PEGylated polymers stabilize hydrophobic phytochemicals and concentrate their payloads within water channels and at device surfaces, thereby increasing local intrabiofilm exposure. For example, curcumin plus amphotericin B co-loaded into porous microparticles improves anti-adhesion and antibiofilm efficacy against *C. albicans* compared with amphotericin B alone, while also reducing amphotericin-associated toxicity; independent datasets report CUR + AMB synergy with FICI = 0.5 and a fourfold reduction in AMB MIC in clinical isolates [20]. Quorum-guided delivery with farnesol-loaded nanoparticles (PLGA and related polymers) achieves deeper penetration and approximately 2-log reductions in cross-kingdom biofilms, and pre-biofilm application suppresses *Candida* biofilm formation more effectively than free farnesol [117, 118]. More broadly, recent reviews on fungal biofilm nanotherapies show that nanocarriers enhance eradication by improving intrabiofilm distribution and enabling co-delivery of adjuvant plus antifungal payloads [119].

### Worked Design Patterns (Actionable Combinations)

Three recurring design patterns illustrate how the mechanistic and quantitative principles outlined above can be implemented in practice. First, membrane-fluidizing phytochemicals such as carvacrol or thymol can be paired with azoles and, when needed, supplemented with an efflux modulator to deepen exposure: a practical workflow is to start with carvacrol (or thymol) plus fluconazole, target FICI ≤ 0.5, and, if synergy is borderline, add a pump-modulating compound such as kaempferol to down-shift CDR1/CDR2/MDR1 expression and re-run checkerboard and sessile time–kill assays, which tends to yield the largest benefits in early- to intermediate-stage biofilms [104, 106]. Second, EPS-opening enzymes can be layered onto azole or polyene backbones by adding β-1,3-glucanase or DNase to a standard regimen: β-1,3-glucanase alone reduces mature biofilm biomass by roughly half and enhances fluconazole or amphotericin B activity, while DNase plus amphotericin B improves killing in XTT and viable-count assays; in both cases, MBEC shifts should be explicitly quantified before and after enzyme addition [109, 120]. Third, co-loaded or sequential nano-delivery can be used to couple pharmacology and spatial targeting by co-encapsulating CUR + AMB or delivering farnesol-containing nanoparticles followed by an azole and then measuring reductions in thickness on CLSM, mechanical softening on AFM, and compression of MBECs, thereby directly aligning formulation choices with the biofilm-aware success criteria defined in Sect. "Quantitative Endpoints and Success Criteria Under Biofilm-Aware Conditions" [20].

## Discussion

### What We Know (Integrated Model)

Biofilm tolerance in *Candida* is an emergent systems property generated by three interconnected layers. At the regulatory level, the master transcription-factor circuit (Bcr1/Efg1/Tec1/Ndt80/Rob1/Brg1 with Flo8) orchestrates yeast–hypha switching, adhesin expression (ALS/HWP), matrix programs, and dispersal, such that perturbations of single nodes only partially reduce biofilm capacity [10, 121, 122]. At the material level, mature communities develop a thick (≈100–500 µm), channelled architecture and an extracellular matrix whose composition centres around ~ 55% protein, 25% carbohydrate, 15% lipid, and 5% nucleic acid, including a mannan–glucan complex that binds antifungal drugs [38, 123]. At the cell-intrinsic level, ATP-binding cassette transporters (CDR1/CDR2), major facilitator transporters (MDR1), and slower physiological states sustain intracellular drug levels below lethal thresholds, while extracellular β-1,3-glucan sequesters azoles and polyenes and enzymatic disruption of glucan delivery (Bgl2/Phr1/Xog1) sensitizes biofilms [6, 83]. Together, these layers explain the MIC–MBEC gap: fluconazole MICs for planktonic *C. albicans* commonly lie within ≤ 0.125–8 µg/mL, whereas MBEC/SMIC values for mature biofilms often require ≥ 256–1,024 µg/mL [5, 84, 124]. Natural compounds contribute when they multi-hit these axes by attenuating TF programs, fluidizing membranes and perturbing sterols, thinning or softening the matrix, and depressing efflux, thereby restoring pharmacodynamic leverage.

### Gaps and Controversies (and How to Close Them)

Despite substantial progress, several mechanistic and translational gaps remain. Regulatory specificity is still elusive because direct chemical binders or modulators of Bcr1, Efg1, Tec1, UME6, and related factors are rare; most evidence comes from pathway-level (cAMP–PKA/MAPK) or phenotypic readouts (adhesins and hyphae), so target engagement should be verified with drug-on ChIP-seq or ATAC-seq and chemoproteomics that benchmark transcription-factor occupancy under exposure, building on existing network foundations [10]. Assay variability in inoculum, media, shear, biofilm age, and endpoints hampers comparability across studies, which argues for a minimal orthogonal panel of CV/XTT, CLSM thickness and live–dead imaging, SEM/AFM mechanics, and MBEC with confidence intervals, leveraging recent methods papers (e.g., iCBiofilm and AFM/rheology reviews) for standardization [33, 125]. Crosstalk between extracellular matrix chemistry and efflux is poorly defined: matrix mannan–glucan clearly shapes host and drug interactions—for example, neutrophil impairment by matrix mannan/glucan—but it is still unknown how EPS composition modulates efflux expression and spatial partitioning, suggesting that spatial omics along periphery-to-core gradients are needed to test causal order [77]. Mixed-species biofilms with bacteria such as *Staphylococcus aureus* further complicate predictions because altered quorum and matrix environments shift the performance of phytochemical/azole combinations, supporting consortia-aware testing strategies [1, 126]. Species coverage is heavily skewed toward *C. albicans* even though non-*albicans* species now account for a large fraction of invasive candidiasis and outbreaks; *C. auris* is a particularly important outlier with multidrug resistance, thin but strongly adherent and highly tolerant biofilms, and efflux- and stress-centred tolerance programs that only partly overlap with the hypha-centric logic of *C. albicans*, so future adjuvant studies should routinely include panels of *C. albicans*, *C. glabrata*, *C. tropicalis*, and *C. auris* with defined resistance phenotypes and report species-stratified synergy metrics, MBEC compression, and virulence readouts rather than extrapolating wholesale from *C. albicans* [14, 15, 61, 62, 64, 65, 68]. On the translational side, solubility, stability, and tolerability remain major bottlenecks, and nanocarriers such as curcumin–amphotericin co-loaded PLGA particles or farnesol-loaded nanoparticles provide one route to increasing intramatrix exposure while reducing toxicity for device- or mucosal indications [20, 118, 127]. Finally, clinical endpoints are still heterogeneous and only loosely connected to quantitative microbiology; bridging SMIC/MBEC and time–kill readouts to device-salvage and mucosal randomized trials, and prospectively registering FICI/Bliss analysis plans, would reduce bias and make model-choice effects in synergy statistics more transparent [110].

### Future Directions (Actionable Roadmap)

Looking ahead, several concrete lines of work emerge from this synthesis. First, regulatory-centric discovery could use dual reporters such as ALS3p–GFP and UME6p–mCherry to triage natural or synthetic hits that invert hypha ↔ yeast programs and then escalate promising compounds to ChIP-seq or related assays to benchmark their impact on the six-transcription-factor circuit [122]. Second, matrix-aware PK/PD studies should quantify intrabiofilm drug gradients using approaches such as LC–MS microdialysis or fluorescent analogues, integrate these measurements into reaction–diffusion models that explicitly incorporate binding to β-glucan and extracellular DNA, and prospectively predict MBEC compression achievable with EPS-targeting enzymes or membrane fluidizers [83]. Third, precision formulation work should focus on pH-responsive, EPS-binding carriers and bioadhesive films tailored for oral and vaginal candidiasis, with in-depth validation of intrabiofilm penetration and mechanical softening by AFM alongside CLSM-based measurements of biofilm thickness [114]. Fourth, adaptive combination design could algorithmically select dual or triple regimens guided by strain-resolved efflux and EPS phenotyping, with the a priori requirement that candidate pairs achieve FICI ≤ 0.5 plus positive Bliss excess across at least three concentration pairs before being considered genuinely synergistic [128]. Finally, host-directed adjunct strategies could leverage matrix-immune insights—for example, the ability of matrix components to suppress neutrophil extracellular traps—to develop β-glucan-exposing antibodies or tuned cytokine cues that promote phagocyte ingress and clearance without triggering immunopathology [77].

### Practical Recommendations (What “Good” Looks Like)

In practical terms, we suggest that “good” biofilm-targeted combinations are those that satisfy the quantitative criteria outlined in Sect. "Quantitative Endpoints and Success Criteria Under Biofilm-Aware Conditions"—namely, robust synergy on biofilm-aware checkerboards, concordant improvements in sessile killing, structural and biomechanical disruption of mature biofilms, and meaningful compression of MBECs toward clinically achievable exposures. Together, these guardrails provide a pragmatic, mechanistically informed framework for comparing natural-compound adjuvants across heterogeneous *Candida* biofilm models and prioritizing combinations with genuine translational promise.

### Limitations of This Review

This is a narrative (not systematic) synthesis. We weighted studies by model validity (clinical isolates; mature biofilms), assay orthogonality, and reporting transparency, but publication bias and method heterogeneity persist; reported ranges (e.g., MBEC, FICI) are representative rather than pooled meta-estimates [125].

## Conclusions

### Scope and Limitations

This is a narrative synthesis; model heterogeneity (species/strain, biofilm age/surface) and endpoint diversity preclude formal pooling, so quantitative ranges here are indicative, not meta-analytic estimates.

### Synthesis vs Objectives

Reframing therapy from killing planktonic cells to dismantling the biofilm state aligns with our objectives and the evidence synthesized here: natural compounds and their adjuvant use act on the axes that sustain biofilms—regulatory circuits, matrix architecture, membranes/sterols, and efflux—thereby translating exposure into killing and narrowing the MIC–MBEC gap. At the molecular level, representative signatures include partial suppression of EFG1/TEC1/BCR1/UME6 outputs—for example, ↓ALS1/ALS3/HWP1, ↓UME6/ECE1, ↑YWP1—which, especially with early-phase dosing, map to reduced biomass/viability (CV/XTT), thinner CLSM architectures, and softer matrix mechanics (SEM/AFM), preventing progression to high-tolerance structure. Concordant falls in adhesion and Sap/phospholipase activity link these shifts to attenuated tissue damage and inflammation, addressing the virulence objective.

### Quantitative Takeaways

Rational pairings—membrane-active phytochemicals + azoles, optionally with efflux or EPS-modifying partners—recurrently show synergy (FICI ≤ 0.5) and ≥ 2–3 log10 sessile time–kill advantages over the best monotherapy, with CLSM/AFM documenting 30–50% decreases in thickness/stiffness; MBECs for mature biofilms shift down from typical high bands (e.g., ≥ 256–512 μg/mL for fluconazole) toward MIC-relevant exposures. (Exact values remain model-dependent and are detailed in the cited exemplars.)

### Path to Translation

Based on this synthesis, we propose four specific, testable statements to guide future work. First, phenylpropanoid and phenolic-monoterpene scaffolds that combine an aromatic ring, a reactive aldehyde or phenolic hydroxyl group, and moderate hydrophobicity are predicted to be key drivers of antibiofilm activity and selective synergy with azoles and polyenes; this hypothesis can be tested by systematically varying these features within cinnamaldehyde/eugenol and thymol/carvacrol analog series and comparing MBIC/MBEC and FICI readouts against matched planktonic MICs. Second, polyphenolic and alkaloid adjuvants that depress efflux capacity or virulence-enzyme output without strong stand-alone fungicidal activity should be prioritised as “silent” potentiators, and evaluated in vitro for their ability to compress MBEC–MIC gaps while maintaining acceptable host-cell toxicity profiles. Third, in vivo validation should focus on a small set of rationally chosen combinations from these clusters (for example, phenylpropanoid- or monoterpene-like adjuvants plus fluconazole or amphotericin B) in device-associated and mucosal candidiasis models, with predefined endpoints including fungal burden, time to clearance, host inflammation and resistance emergence. Fourth, translational programmes should explicitly couple these mechanistic and in vivo studies to pharmacokinetic/pharmacodynamic and formulation work—defining the local concentrations and dwell times that are realistically attainable with topical, lock or surface-directed delivery, and using response-surface or similar designs to optimise drug–adjuvant ratios rather than testing single, arbitrary dose combinations.

## Supplementary Information

Below is the link to the electronic supplementary material.

Supplementary file1 (DOCX 18 KB)

## Data Availability

All data generated or analyzed during this study are included in this published article.
