# Supplementary material for: Natural-Compound Adjuvants Dismantle Candida Biofilms: Mechanisms, Design Rules, and Biofilm-Aware Pharmacology
Source: Curr Microbiol. 2026 Jan 12;83(2):131. doi: 10.1007/s00284-025-04713-0 (PMC12795885; doi:10.1007/s00284-025-04713-0)
Supplement: Supplementary file 1 — Supplementary file1 (DOCX 18 KB) [file 284_2025_4713_MOESM1_ESM.docx]

**Methods S1. Search strategy, eligibility criteria, data extraction, quality appraisal, and evidence synthesis**

**S1.1 Information sources and coverage**

We searched PubMed, Scopus, and Web of Science and performed backward and forward citation chasing to identify additional relevant studies. Records were limited to English-language publications or English abstracts with sufficient methodological detail to assess biofilm-related outcomes. Preprints were consulted only when a peer-reviewed version was unavailable or when the preprint and the final article were materially identical. Reviews and guidelines were used selectively to orient the literature, terminology, and broader context; primary studies provided the evidentiary basis for all mechanistic and translational conclusions. A final comprehensive search across all information sources was run on 23 August 2025.

**S1.2 Search strategy**

Database-specific Boolean search strings combined organism terms, phenotype/structure terms, regulatory pathways, biofilm pharmacology endpoints, and natural-compound/combination terms. No study-type filters were applied at the search stage in order to avoid missing mechanistic work; eligibility criteria were applied during screening.

A representative PubMed query was:

• (Candida OR "Candida albicans" OR "Candida glabrata" OR "Candida tropicalis" OR "Candida auris") AND

• (biofilm OR "extracellular matrix" OR hypha* OR filament*) AND

• (BCR1 OR EFG1 OR TEC1 OR UME6 OR "Ras1" OR cAMP OR PKA OR MAPK OR Hog1 OR Mkc1 OR quorum OR farnesol OR tyrosol) AND

• (efflux OR CDR1 OR CDR2 OR MDR1 OR SNQ2 OR MBEC OR "biofilm eradication concentration") AND

• (natural compound* OR phytochemical* OR "essential oil*" OR synergy OR "combination therapy" OR adjuvant)

Minor variants of this query were adapted to database-specific syntax and used during snowballing to capture additional mechanistic or translational studies.

**S1.3 Eligibility criteria**

**Inclusion criteria**

Studies were eligible if they addressed at least one of the following primary objectives:

• Regulatory targeting: modulation of master transcription factors (e.g., BCR1, EFG1, TEC1, UME6), upstream signaling pathways (Ras1–cAMP–PKA; MAPK/Hog1/Mkc1), quorum-sensing cues (farnesol, tyrosol), or epigenetic/RNA-mediated regulation relevant to *Candida* biofilms.

• Phenotypic disruption: quantitative biofilm outcomes such as biomass (crystal violet), metabolic viability (XTT/resazurin), biofilm eradication concentrations (MBEC or related measures), thickness and architecture (e.g., CLSM z-stacks), extracellular matrix composition or ultrastructure (SEM/TEM/AFM), and hyphal indices.

• Virulence traits: adhesion assays, protease/phospholipase activity, or host-interaction surrogates linked to biofilm formation or maintenance.

• Synergy: combinations of natural compounds with azoles, echinocandins, or polyenes, quantified using established models (e.g., FICI, Bliss independence, Loewe additivity, highest single agent [HSA], or ZIP).

Eligible study designs included in vitro biofilm models (mono- or mixed-species, preferably on clinically relevant materials), in vivo animal models, and clinical or observational studies reporting biofilm-relevant outcomes.

**Exclusion criteria**

We excluded:

• Studies of non-*Candida* biofilms without a *Candida arm*;

• Planktonic-only antifungal tests not linked to any biofilm endpoint;

• Opinion pieces, narrative overviews, or guidelines lacking primary data;

• Studies with insufficient methodological detail to appraise biofilm endpoints or regulatory claims;

• Non-English records without adequate English-language summaries.

**S1.4 Study selection and de-duplication**

Records retrieved from all databases were first de-duplicated. Screening proceeded in two passes:

1. Title and abstract screening to remove clearly off-topic items.

2. Full-text assessment against the predefined inclusion and exclusion criteria.

Given the narrative design of this review, screening and eligibility decisions were performed by the author with a confirmatory second pass to reduce oversight. Discrepancies were resolved by re-checking the source texts against the prespecified criteria. Because this is not a systematic review, we did not construct a PRISMA flow diagram.

**S1.5 Data items and extraction**

From each eligible study, we extracted:

• Organisms and strains, including species, strain identifiers, and whether isolates were clinical or laboratory-adapted;

• Biofilm models, including substrate or surface (e.g., polystyrene, silicone, catheter, denture acrylic), media, shear conditions (static versus flow), temperature, time points, and biofilm age at treatment;

• Assay methods, including biomass/viability readouts (crystal violet, XTT, resazurin, CFU), imaging/biophysical methods (CLSM, SEM, TEM, AFM), and MBEC platforms and surfaces used;

• Regulatory readouts, including qPCR, RNA-seq, ChIP or other transcriptional/protein-level measurements, quorum-sensing manipulations, and epigenetic marks where reported;

• Virulence metrics, such as adhesion indices, protease and phospholipase activity, and other host-interaction surrogates;

• Synergy metrics, including the model used (FICI with thresholds, Bliss excess, Loewe deviation, HSA, ZIP), together with checkerboard, time-kill, or response-surface design characteristics;

• Pharmacological parameters, including MICs (CLSI M27 where available), MFC/MIC ratios, MBEC or SMIC values, and exposure regimens for in vivo or clinical studies.

Where necessary, concentrations were harmonized to µg/mL and time to hours for comparability across studies. Synergy matrices were annotated with the reference model (e.g., Bliss versus Loewe) and the experimental design (checkerboard, time-kill, or response-surface).

**S1.6 Quality appraisal (narrative, design-appropriate)**

Because formal risk-of-bias tools are not standardized for in vitro biofilm studies, we adopted a domain-based checklist to weight the evidentiary strength of each study:

• Model validity: use of clinical isolates; biofilm age consistent with “mature” claims (typically ≥24–48 h); and use of clinically relevant surfaces or conditions where appropriate;

• Assay orthogonality: reliance on more than one readout (e.g., crystal violet and XTT plus microscopy) rather than single-assay claims;

• Controls and comparators: inclusion of vehicle/solvent controls, appropriate reference drugs, and concentration ranges spanning sub-MIC to supra-MBEC levels;

• Replicate depth and statistics: reporting of biological and technical replicates, effect sizes with measures of variance, and appropriate statistical analyses;

• Mechanistic support: transcript, protein, or imaging evidence consistent with proposed regulatory or structural targets;

• Reporting transparency: clear documentation of strain IDs, media composition, inoculum density, incubation conditions, and treatment protocols.

For animal or clinical studies, additional considerations included internal validity (randomization and blinding where feasible), outcome definitions, and justification of dosing regimens. These quality domains did not generate numeric scores but informed the narrative weighting of findings (e.g., highlighting orthogonally validated results and flagging claims that rested on weaker methods).

**S1.7 Evidence synthesis and handling of heterogeneity**

**Rationale for not performing a meta-analysis**

Experimental heterogeneity across *Candida* biofilm studies violates core assumptions for quantitative pooling. Species, strains, and morphotypes differ; biofilm age (e.g., 6–72 h) and surfaces (polystyrene, silicone, catheter, denture acrylic) vary widely; endpoints are operationalized using non-standardized measures (MBIC, SMIC50/80, MBEC, sessile time-kill) and diverse assays (crystal violet, XTT, resazurin, CFU). Synergy is quantified using different models (FICI, Bliss, Loewe, HSA, ZIP) under disparate biofilm conditions. Concentrations and units are rarely harmonized, and effect sizes often lack comparable variance measures. Under these conditions, pooled effect estimates would be potentially misleading.

**Approach to synthesis**

We therefore adopted a semi-quantitative narrative synthesis. This approach emphasizes:

(i) the direction and consistency of effects across independent studies;

(ii) typical magnitude ranges rather than single-point estimates (e.g., azole–terpenoid combinations frequently shift biofilm eradication requirements from high-hundreds of µg/mL toward double-digit exposures; checkerboards performed under biofilm conditions often report synergy-range FICI values); and

(iii) triangulation across orthogonal readouts (e.g., concordant changes in microscopy/biophysics and biomass/viability assays).
